# A Report of Two Cases of Embryonal Rhabdomyosarcoma: Diagnostic Insights From Pathology

**DOI:** 10.7759/cureus.80498

**Published:** 2025-03-12

**Authors:** Preeti R Doshi, Rachana Lakhe, Vishal Panjvani, Manjiri Karandikar, Reena Bharadwaj

**Affiliations:** 1 Pathology, Bharati Vidyapeeth (Deemed to be University) Medical College, Pune, Pune, IND

**Keywords:** embryonal, immunohistochemistry, pediatrics, rhabdomyosarcoma, urologic oncology

## Abstract

Rhabdomyosarcoma (RMS) is a family of malignant soft tissue tumors derived from undifferentiated mesoderm that fails to differentiate into skeletal muscle. Embryonal RMS (ERMS) is the most common subtype of RMS in children and adolescents, particularly those under 10 years of age. It primarily arises in the head and neck region, genitourinary tract, or extremities. Histologically, it resembles developing skeletal muscle with anaplastic features. Diagnosis relies on a combination of imaging, histopathology, immunohistochemistry, and molecular studies, with specific genetic alterations noted in the literature. Effective treatment of pediatric RMS cases requires multimodal therapy, including surgery, chemotherapy, and radiotherapy, to achieve a favorable prognosis. We report two cases of ERMS diagnosed within a four-month period, where immunohistochemistry and molecular studies contributed to the diagnosis.

## Introduction

Rhabdomyosarcoma (RMS) is a malignant soft tissue tumor of skeletal muscle origin first recognized by Weber in 1854 [1]. It arises from immature striated skeletal muscle and can occur at any age or location on the body. The most common primary site for RMS is the orbit within the head and neck region [2]. In the oral cavity, the tongue is most frequently affected, followed by the soft palate, hard palate, and buccal mucosa. RMS affects approximately 6.4 cases per million newborns and infants each year, with about 5% of cases originating in the prostate and urinary bladder [3].

RMS exhibits biochemical and behavioral characteristics of skeletal muscle differentiation and is derived from the undifferentiated mesoderm. A notable cytogenetic feature is the loss of heterozygosity of chromosome 11p15.5, with trisomy 8 also commonly identified. Lymph node metastasis is more likely to occur in tumors originating from genitourinary sites, although infant genitourinary embryonal RMS (ERMS) generally has a better prognosis [4].

Unfortunately, RMS has a low survival rate. The most recent Children’s Oncology Group (COG) intermediate-risk study reported a four-year event-free survival rate of only 63% [5]. Microscopically, RMS appears as small, rounded, spindled cells with varying degrees of eosinophilic cytoplasm [6].

We present two cases of ERMS diagnosed within a four-month period.

## Case presentation

Case 1

**Figure 1:**
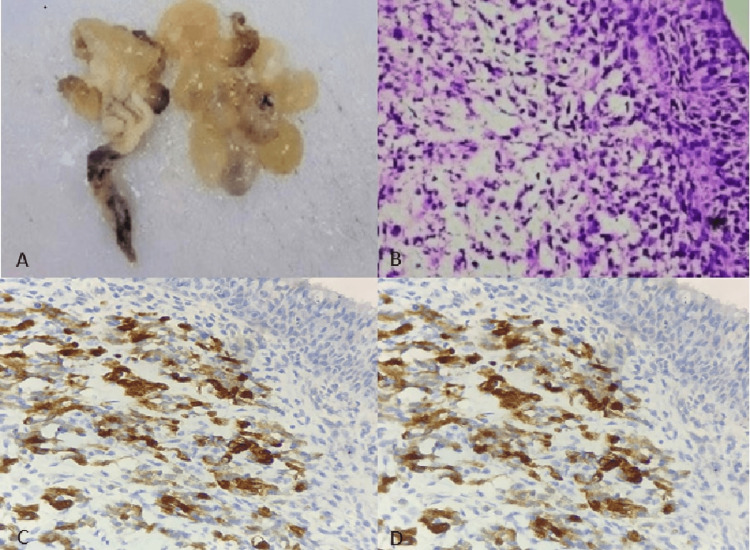
Case 1: (A) Gross image displaying a glistening, gelatinous, fleshy nodular appearance. (B) Cambium layer composed of immature round cells observed subepithelially, accompanied by numerous spindle cells (40x). (C) Tumor cells demonstrating cytoplasmic desmin positivity (40x). (D) Tumor cells showing vimentin positivity (40x).

An eight-month-old male child presented with a mass in the urinary bladder. Imaging by CT scan showed multiple lobulated, variably sized cystic lesions suggestive of a neoplastic origin. On gross examination, we received multiple polypoidal, grape-like pieces from the excised urinary bladder mass (Figure 1A). Histology revealed urothelial lining with a cambium layer consisting of immature round cells located subepithelially. Numerous spindle cells with scant mitotic activity were scattered within the myxoid stroma (Figure 1B). Immunohistochemistry demonstrated positivity for desmin, vimentin, and MyoD1 (Figure 1C, 1D), while fluorescent in situ hybridization showed that the PAX3-FOXO1 fusion gene was negative.

Case 2

**Figure 2:**
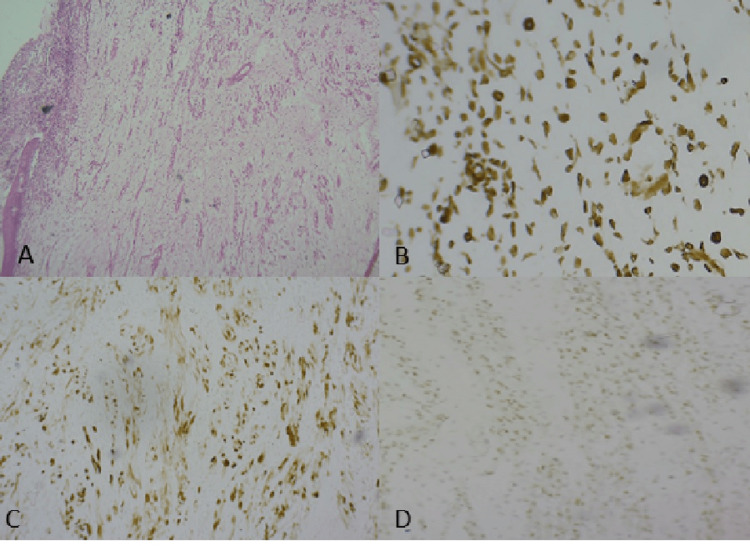
Case 2: (A) Cambium layer composed of immature round cells observed subepithelially, along with numerous spindle cells (40x). (B) Tumor cells demonstrating cytoplasmic desmin positivity (40x). (C) Tumor cells showing vimentin positivity (40x). (D) Tumor cells exhibiting MyoD1 positivity (40x).

A one-year-old male child presented with a mass in the urinary bladder. Imaging by CT scan showed an ill-defined, hypolobulated mass measuring 3.6 × 2.4 × 2.1 cm located at the posterior aspect of the bladder base. On gross examination, multiple grape-like pieces from the bladder mass were excised. Histopathological examination revealed hypocellular and cellular areas containing round, spindle, and tadpole-shaped cells within a myxoid stroma. A cambium layer was noted (Figure 2A). Immunohistochemistry demonstrated positivity for desmin, vimentin, and MyoD1 (Figure 2B, 2C, 2D), while fluorescent in situ hybridization showed that the PAX3-FOXO1 fusion gene was negative.

## Discussion

Children between the ages of one and four years have the highest incidence of RMS, followed by those between 10 and 19 years. RMS in children most commonly develops in the genitourinary tract. ERMS is the most frequently observed pediatric genitourinary tumor, with an incidence of approximately 4.5 cases per million children and adolescents per year [7].

To differentiate RMS from other similar diagnoses, such as Ewing’s sarcoma, spindle cell sarcoma, and osteosarcoma, a thorough histological and immunohistochemical analysis is essential [8].

Histologically, RMS is classified into two main subgroups: ERMS, which accounts for 60-70% of cases [9], and alveolar RMS (ARMS), which comprises 20-30% of cases [10]. Several studies have identified the presence of a FOXO1 fusion as an independent negative prognostic factor in localized RMS [11-13].

A characteristic genetic alteration in pediatric aRMS is the PAX3-FOXO1 fusion gene. This stable reciprocal translocation between chromosomes 2 and 13 results in an in-frame fusion of the DNA-binding domain of PAX3 with the transactivation domain of FOXO1. The expression of this fusion gene is associated with a poor prognosis, particularly in cases involving metastasis [11].

Effective treatment of ERMS requires multimodal therapy, including surgery, chemotherapy, and radiotherapy. Despite significant advancements, the likelihood of curing children with widespread metastatic or recurrent disease remains extremely low [4]. Current risk stratification for RMS prognosis considers factors such as clinical group, tumor site, size, invasiveness, and lymph node status [4].

In both of our presented cases, similar features were observed, including a cambium layer consisting of immature round cells located subepithelially, numerous spindle cells, scattered rhabdomyoblasts, and positive staining for desmin, vimentin, and myogenin. The absence of FOXO1 gene rearrangements differentiates poorly differentiated ERMS from solid ARMS in both cases.

## Conclusions

ERMS is a rare but significant pediatric malignancy. Its presentation is not always classic, making diagnosis challenging. A high index of suspicion, combined with thorough clinical and pathological correlation, is essential for prompt diagnosis, early treatment, and better outcomes. These two cases highlight the critical role of histopathology in diagnosing ERMS, emphasizing the importance of combining histopathological analysis, immunohistochemistry, and molecular studies to distinguish it from other soft tissue sarcomas. Moreover, molecular studies can provide valuable insights into the disease process, aiding prognosis and guiding treatment strategies. Continued advancements in diagnostic techniques are crucial for improving outcomes and quality of life for patients with RMS.
